# Liver Granulocytic Sarcoma With Megakaryocytic Differentiation: A Rare Extra Medullary Involvement That Warrants Liver Biopsy for Prompt Diagnosis

**DOI:** 10.7759/cureus.16366

**Published:** 2021-07-13

**Authors:** Hira Chaudhary, Haytham Aboushi, Jeremy Minkowitz, Jodi-Ann Edwards, Daniel Beltre, Priyanka Parmar, Igal Breitman, Carol Luhrs, Samy I. McFarlane

**Affiliations:** 1 Department of Medicine, State University of New York (SUNY) Downstate Health Sciences University, Brooklyn, USA; 2 Department of Medicine, State University of New York (SUNY) Downstate Medical Center, Brooklyn, USA; 3 Department of Surgery, State University of New York (SUNY) Downstate Health Sciences University, Brooklyn, USA; 4 College of Medicine, State University of New York (SUNY) Downstate Health Sciences University, Brooklyn, USA; 5 Department of Hematology and Oncology, State University of New York (SUNY) Downstate Health Sciences University, Brooklyn, USA; 6 Department of Internal Medicine, State University of New York (SUNY) Downstate Health Sciences University, Brooklyn, USA

**Keywords:** granulocytic sarcoma, myelodysplastic syndrome, acute myeloid leukemia, myeloid sarcoma, liver biopsy

## Abstract

Granulocytic sarcoma (GS) is an extramedullary manifestation of acute myeloid leukemia (AML), myelodysplastic syndrome (MDS) or myeloproliferative neoplasms. The diagnosis depends on morphology, immunohistochemistry and flow cytometry. An unusual location of this tumor may mask its primary source, therefore, a strategy involving immediate symptom control, and investigation is crucial in preventing clinical deterioration. We present a case of a 53-year-old man who initially presented with tumor lysis syndrome and transaminitis, with a subsequent CT Scan that revealed multiple liver lesions. This case describes a rare clinical entity of granulocytic sarcoma as multiple hypoattenuating liver lesions mimicking metastatic disease in its radiographic appearance. Since the imaging features of hepatic masses are nonspecific, and considering the aggressive nature of AML with concomitant tumor lysis syndrome, a confirmatory prompt biopsy should routinely be considered.

## Introduction

Granulocytic sarcoma (GS), also known as myeloid sarcoma (MS), and chloroma originates from immature cells of myeloid progenitor series. The commonly involved extramedullary sites of acute myeloid leukemia (AML) are skin, central nervous system, lymph node, bone, soft tissue, and testis [1]. Along with leukemia cutis, meningeal involvement, and gingival hypertrophy, it is a well-defined extramedullary manifestation of AML [2]. It can present as a solitary or multifocal lesion and is an indicator of poor clinical outcome. It may be diagnosed prior to, after, or concomitant with the diagnosis of AML or may present de-novo [3,4]. The overall incidence is 2.5%-9% of patients with AML and less than 1% of patients present with extramedullary disease prior to the diagnosis of AML [5,6]. Less commonly, it can also be seen in patients with underlying myelodysplastic syndrome (MDS), AML, or other myeloproliferative diseases as suspected in our case. Although granulocytic sarcoma can arise in any area of the body, tumors with megakaryoblastic differentiation in the liver are not often reported and may be a sequela of transformation to a myeloproliferative disorder.

## Case presentation

**Figure 1:**
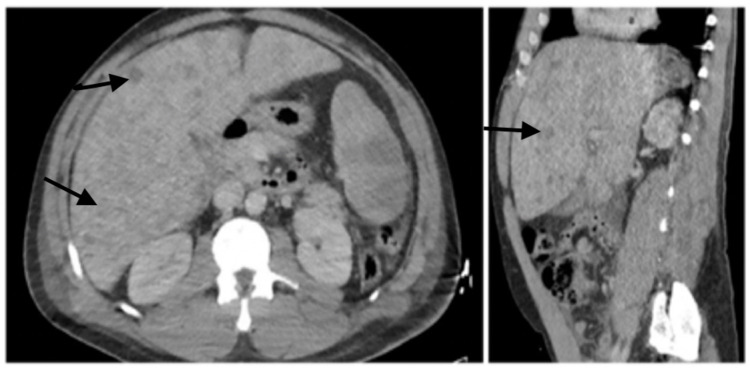
CT abdomen/pelvis scan with the presence of multiple hypodense liver lesions.

A 53-year-old man with a history of hypertension presented to our institution complaining of one week of constipation and dyspnea. Initial assessment revealed temperature 96.7 F, BP 104/75 mmHg, heart rate 112 bpm, respiratory rate 22 breaths/min, and saturation of 97% on room air. Physical exam was remarkable for abdominal distension and reduced breath sounds in the left lower lung. Labs revealed metabolic abnormalities including hyperkalemia, hyperuricemia, acute renal injury, consistent with tumor lysis syndrome. Liver tests showed alkaline phosphatase of 394 U/L, aspartate transaminase (AST) 265 U/L, alanine aminotransferase (ALT) 230 U/L, total bilirubin 3.2 mg/dL. Coagulation studies showed international normalized ratio (INR) to 1.7, prothrombin time (PT) 21.1 s, partial thromboplastin time (PTT) 28.1 s, and a D-dimer of 9138 ng/mL. Lactate dehydrogenase (LDH) 1338 U/L, C-reactive protein (CRP) 206 mg/L, and ferritin of 7561 ng/mL. UA was unremarkable. His CBC revealed leukocytosis (WBC 24.28 K/uL) with neutrophilic predominance (17.8 K/uL) and 9% bands with 2% atypical lymphocytes, 6% monocytes, 1% myelocytes, and mild thrombocytopenia (PLT 123 K/uL.) CT chest, abdomen, and pelvis with contrast revealed a left lower lobe pleural effusion hepatosplenomegaly (liver 25.5 cm, Spleen 21.2 cm) with hypoattenuating liver and splenic lesions, and ascites (Figure 1).

**Figure 2:**
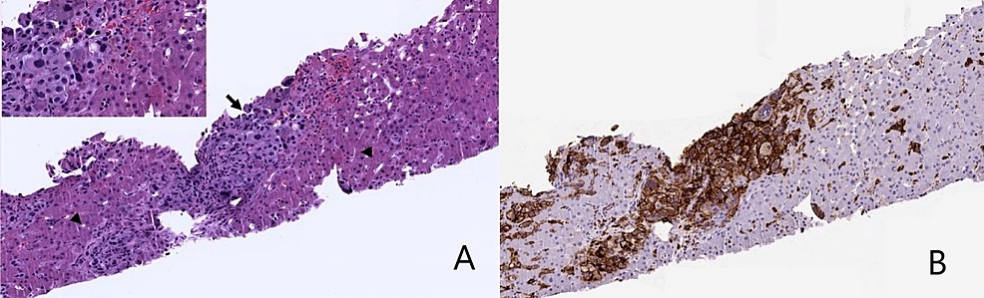
Liver biopsy showing tumor cells that are CD43 positive. A: Core biopsy of liver showing immature megakaryocytes (arrow) flanked by benign hepatocytes (arrowheads) accentuated on higher power; B: CD43 immunohistochemistry staining showing strong and diffuse membranous expression on immature leukemic megakaryocytes surrounded by hepatocytes.

Patient was initially managed with aggressive fluid resuscitation and broad-spectrum intravenous antibiotics. His hyperuricemia was refractory to rasburicase. He subsequently developed oliguric kidney failure requiring hemodialysis. A subsequent peripheral blood smear showed a white count of 47.25 K/uL. No blasts were identified. On day 6 of hospitalization, he went into cardiac arrest, with no return of spontaneous circulation after the ACLS code. Liver biopsy that was performed a day before he died, revealed cords of hepatocytes with dilated sinusoids packed with immature hematopoietic cells. Within the neoplastic cells were scattered large atypical cells that resembled atypical megakaryocytes. Immunohistochemical stains were performed and blasts were strongly positive for CD43, CD61, CD31 and weakly stained for CD71, lymphoid markers (CD45) and CD68. They were negative for glycophorin, myeloperoxidase, CD34, and epithelial markers (EMA, AE1/AE3). These immunophenotypic features were consistent with the diagnosis of granulocytic sarcoma with megakaryocytic differentiation (Figure 2). The patient did not have a previous history of any myeloproliferative neoplasms or acute leukemia. Bone marrow biopsy could not be performed as the patient had passed away.

## Discussion

GS also known as MS can occur in four clinical settings. It can occur (1) in patients with diagnosed AML in the active phase of the disease; (2) in patients with chronic myeloproliferative disorder or myelodysplastic syndrome, it can manifest as the first sign of blastic differentiation; (3) in patients as an initial manifestation in AML; (4) in healthy individuals in whom AML occurs weeks, months, years later or rarely never occurs [6,7]. 75% of patients were middle-aged based on the study of primary GS conducted on patients at the MD Anderson Cancer Center [6]. Interestingly, it can be detected before any clinical signs or evidence of leukemia or other neoplasms. Typically, GS presents with myeloblastic, monoblastic or myelomonocytic morphology, but GS with megakaryoblastic differentiation is rarely witnessed and reported in the literature [7]. Our patient presented with hepatosplenomegaly with multiple liver masses without any previous history of myeloproliferative neoplasm (MPN) or acute leukemia. Presence of clusters of blasts with megakaryocytic differentiation in the extramedullary site, i.e., liver could represent blastic transformation of extramedullary hematopoiesis, secondary to MPN including myelofibrosis; however, there was no bone marrow biopsy to confirm the primary diagnosis. Based on the clinical evidence of lack of blasts in peripheral smear, MPO negative cells, the leukemic progression was less likely, and the main differential diagnosis could be primary presentation of granulocytic sarcoma with megakaryocytic differentiation in the liver.

Diagnosis is largely dependent on tissue biopsy which mostly consists of infiltration by myeloid precursor cells. Immunohistochemistry, flow cytometry, and molecular analysis further help with definitive diagnosis [7,8]. The importance of cytogenetics in myeloid sarcoma is not fully established but it seems to be a promising technique to guide treatment options as those culprit mutations can be targeted. CD31, CD34, CD61, and Factor VIII are some megakaryocytic markers for early precursor stages [8]. In our case, neoplastic cells were positive for CD61, CD31, CD43, and negative for MPO potentially indicating megakaryocytic differentiation and fairly excluded other possible entities, as immunopositivity for myeloperoxidase is specific for granulocytic differentiation [4]. Next step is a bone marrow biopsy, which is recommended to investigate the underlying hematological malignancy. Unfortunately, our patient had died of complications before a marrow biopsy could be performed. Although GS can develop in any organ, the involvement of the hepatobiliary system is very unusual, therefore it presents as a diagnostic challenge especially in patients with no previous leukemic disease as predicted in our patient. Hence a liver biopsy is of great help. Furthermore, PET-CT is useful in early detection when the disease is clinically occult, and also allows disease staging and treatment monitoring [9].

Treatment modalities consist of radiotherapy, surgery, chemotherapy, and stem cell transplantation [7]. There is no consensus on GS treatments as more trials are required to establish treatment guidelines. The recommended regimen is systemic chemotherapy using AML-induction protocols such as cytarabine and idarubicin, even in non-leukemic disease due to the higher rate of progression to AML [10]. Surgery can be considered for debulking before starting the chemotherapy. Regarding hematopoietic stem cell transplantation, there are no prospective trials evaluating its role in isolated MS, but some retrospective studies show promising results with non-relapsing rate of 17% and longer overall survival, both in isolated MS and MS with AML [7,10]. It is therefore encouraged to consider allogeneic bone marrow transplantation in relapsed patients and in patients that achieve their first remission. Recent advances in genetic profiling could be a game changer in terms of outcomes and prognosis, and may enable the development of novel targeted therapies [6,7]. These agents include fms-like tyrosine kinase (FLT3) inhibitors, farnesyltransferase inhibitors (FTIs), histone deacetylase inhibitors, and DNA methyltransferase inhibitors [10]. To sum things up, more prospective multicenter controlled trials are needed to formulate ideal treatment decisions and investigate the role of novel targeted therapies.

## Conclusions

In the evaluation of a hepatic mass, myeloid sarcoma should be considered in the differential diagnosis. We highlight the importance of occurrence of GS in the liver, tumor lysis syndrome indicating high tumor burden without AML features, and early diagnosis to prevent acute clinical deterioration. It is pivotal for physicians to familiarize themselves with such unconventional presentation of GS which is indeed multifaceted and can be mistaken for a variety of other malignancies leading to delay in diagnosis and treatment.

## References

[1] Rajaretnam N, Malcolm P, Aroori S (2019). Granulocytic sarcoma: an uncommon cause of systemic inflammatory response syndrome. Clin Case Rep.

[2] Lee JY, Lee WS, Jung MK (2007). Acute myeloid leukemia presenting as obstructive jaundice caused by granulocytic sarcoma. Gut Liver.

[3] Abu-Zeinah GF, Weisman P, Ganesh K (2016). Acute myeloid leukemia masquerading as hepatocellular carcinoma. J Gastrointest Oncol.

[4] Li JM, Liu WP, Zhang MH (2006). Clinicopathologic and immunophenotypic analysis of myeloid sarcoma. Chin J Pathol.

[5] Agarwal A, Dadu T, Bhalla VP, Malhotra V (2019). Myeloid sarcoma of bile ducts presenting as obstructive jaundice-a case report. Indian J Pathol Microbiol.

[6] Wu HY, Liu L, Gu L, Luo YH (2019). Clinical characteristics and management of primary granulocytic sarcoma of the breast: A case report. Medicine.

[7] Yilmaz AF, Saydam G, Sahin F, Baran Y (2013). Granulocytic sarcoma: a systematic review. Am J Blood Res.

[8] Obiorah IE, Ozdemirli M (2018). Myeloid sarcoma with megakaryoblastic differentiation presenting as a breast mass. Hematol Oncol Stem Cell Ther.

[9] Tomasian A, Sandrasegaran K, Elsayes KM, Shanbhogue A, Shaaban A, Menias CO (2015). Hematologic malignancies of the liver: spectrum of disease. Radiographics.

[10] Magdy M, Abdel Karim N, Eldessouki I, Gaber O, Rahouma M, Ghareeb M (2019). Myeloid sarcoma. Oncol Res Treat.

